# Role of β-Arrestin 2 in the antinociceptive and side effect profile of morphine and the novel mu opioid receptor agonists, kurkinorin and kurkinol

**DOI:** 10.1038/s41386-025-02214-z

**Published:** 2025-09-03

**Authors:** Ross van de Wetering, Amy F. Alder, Andrew Biggerstaff, Katya Sellen, Dan Luo, Rachel S. Crowley, Thomas E. Prisinzano, Bronwyn M. Kivell

**Affiliations:** 1 School of Biological Sciences, Centre for Biodiscovery, Victoria University of Wellington, Wellington, 6012 New Zealand; 2 Department of Pharmaceutical Sciences, University of Kentucky, Lexington, Kentucky 40506 USA; 3 Department of Medicinal Chemistry, School of Pharmacy, The University of Kansas, 1251 Wescoe Hall Drive, 4070 Malott, Lawrence, Kansas 66045 USA

**Keywords:** Drug discovery, Neuroscience

## Abstract

The development of safer mu opioid receptor (MOR) agonists with reduced side effects is a key focus of pain research. Some studies have suggested that MOR agonists with reduced β-arrestin 2 (βArr2) signaling (i.e. G-protein biased agonists) may have greater therapeutic windows. However, there have been a several conflicting reports, and it is not clear what role, if any, βArr2 signaling plays in MOR-mediated analgesia, tolerance, or side effects. Therefore, we used βArr2 knockout mice to systematically investigate the causal role of βArr2 signaling in antinociception, antinociceptive tolerance, respiratory depression, constipation, and reward induced by morphine and the two novel MOR agonists, kurkinorin and kurkinol. Kurkinorin and kurkinol exhibited potent antinociceptive effects that were reversed by MOR knockout. Unlike morphine or kurkinorin, our most G-protein biased agonist, kurkinol, showed no significant tolerance after seven days of ~2×ED_50_ dosing. However, in a chemotherapy-induced neuropathic pain model, all three compounds were ineffective after 20 days of ~ED_50_ dosing, indicative of tolerance. All compounds exhibited significant MOR-dependent side effects, though kurkinorin had reduced gastrointestinal and respiratory depressive effects compared to morphine despite exhibiting less G-protein bias. Knockout of βArr2 significantly increased antinociceptive potency for morphine and kurkinorin but not kurkinol, and otherwise had no significant impact on tolerance or any side effect tested. These results largely suggest that βArr2 signaling does not drive MOR-mediated antinociceptive tolerance, respiratory depression, constipation, or reward and do not support the development of G-protein biased compounds as a broadly effective strategy to reduce side effects.

## Introduction

The mu opioid receptor (MOR) is the primary target for many clinically used analgesics such as morphine, fentanyl, and oxycodone. While highly effective for severe acute pain, these MOR agonists have significant on-target side effects including respiratory depression, high abuse liability, tolerance, and constipation, which limit their safety and utility in managing chronic pain [1]. The over reliance on these drugs for pain management, coupled with their widespread illicit misuse, has fueled the ongoing opioid crisis, where more than 60,000,000 people globally struggle with their addictive effects, and over 100,000 people die every year due to overdose [2].

It has been proposed that the development of safer MOR agonists can be achieved by exploiting the concept of signaling bias (also known as functional selectivity or biased agonism) [3–6], whereby ligands can induce unique receptor conformations that differentially impact intracellular signaling pathways. The MOR is a G-protein coupled receptor (GPCR) that signals via G_αi/o_. MOR activation also recruits β-Arrestin (βArr) scaffolding proteins that primarily serve to regulate subsequent GPCR signaling but can also independently activate intracellular signaling cascades. Activation of βArr2 specifically has been suggested to mediate some of the adverse side effects of MOR agonists, including analgesic tolerance, respiratory depression, and constipation [3–10]. However, recent research been unable to replicate these findings, possibly due to the mixed C57BL/6 × 129SvJ strain of mice used, and efforts to increase therapeutic windows by developing novel, G-protein biased MOR agonists with minimal βArr2 signaling has seen little success [11–13]. These inconsistent findings highlight the need for a systematic study into the causal role of βArr2 signaling in MOR-mediated antinociception and side effects.

**Fig. 1:**
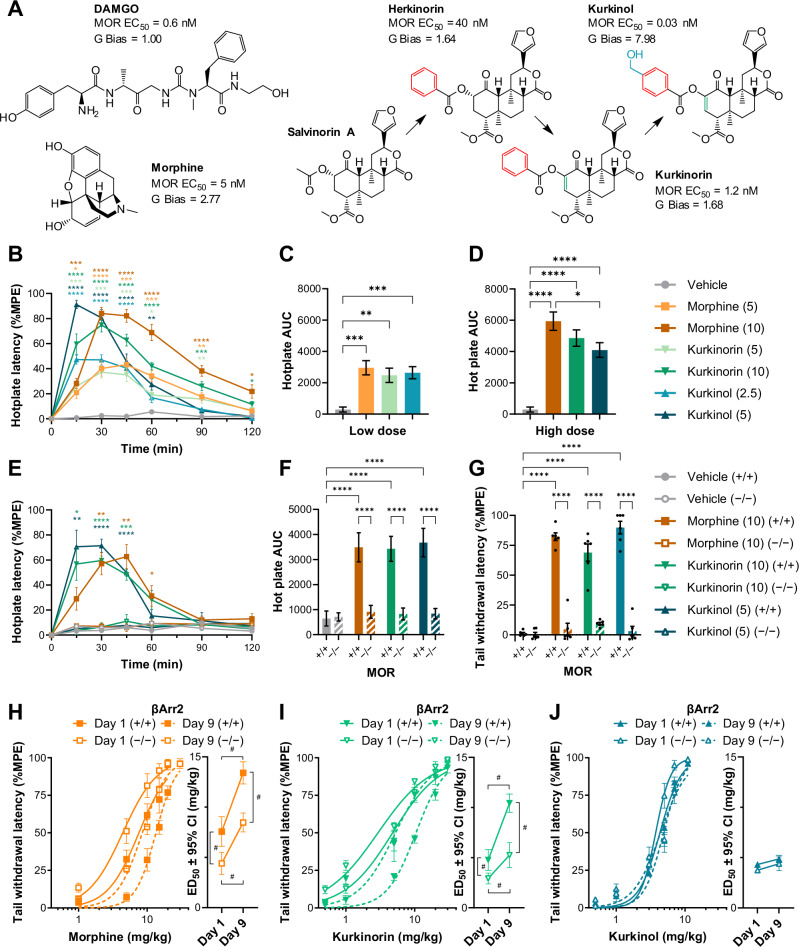
Kurkinorin and kurkinol have MOR-dependent antinociceptive effects similar to morphine, but with reduced tolerance, while knockout of βArr2 increases antinociceptive potency for some drugs but has no effect on the development of tolerance. **A** Chemical structures of kurkinorin, kurkinol, and other relevant compounds with in vitro MOR EC_50_ and G-protein bias data (relative to DAMGO, values > 1 indicate G-protein bias) calculated from Crowley et al. 2016, 2020. Colors represent structural modifications. Time-dependent **B** and overall (AUC; **C, D**) antinociceptive effects of morphine, kurkinorin, and kurkinol in the hotplate assay in male and female mice (pooled). Time-dependent **E** and overall (AUC; **F**) antinociceptive effects of morphine, kurkinorin, and kurkinol in the hotplate assay in MOR knockout male mice. **G** Antinociceptive effects of morphine, kurkinorin, and kurkinol in the warm-water tail withdrawal assay in MOR knockout male mice. Cumulative dose response curves and calculated ED_50_ values (± 95% confidence intervals) for morphine **H**, kurkinorin **I**, and kurkinol **J** in the warm-water tail withdrawal assay in βArr2 knockout male mice before (day 1) and after (day 9) seven days of treatment with a ~ 2×ED_50_ dose of drug. Drug doses are mg/kg. Data are presented as mean ± SEM unless otherwise stated. *n* = 10–12/treatment **B–D**, *n* = 6/treatment/genotype (E-G), *n* = 5–6/treatment **H–J**. *p < 0.05, **p < 0.01, ***p < 0.001, ****p < 0.0001 compared to vehicle treatment or as indicated, one or two-way ANOVA. ED_50_ values calculated by non-linear regression. ^#^95% confidence intervals.

To this end, we investigated two novel and unique MOR agonists, kurkinorin and kurkinol. These compounds are derived from salvinorin A/herkinorin, and show potent activity and selectivity at the MOR, with varying G-protein signaling bias (Fig. 1A) [14, 15]. Initial in vivo work showed that these compounds have significant antinociceptive effects with reduced tolerance compared to morphine in the warm-water tail withdrawal assay [14, 15]. In the current study, we further evaluated the effects of kurkinorin and kurkinol in comparison to morphine using both acute and chronic pain models. We also assessed various side effects, including respiration, constipation, and reward, and investigated the role of βArr2 signaling using βArr2 knockout mice.

## Materials and methods

### Animals

Male and female, 8–12-week-old, C57BL6J, MOR knockout (B6.129S2-OPRM1tm1Kff/J, stock no. 007559, The Jackson Laboratory), or βArr2 knockout (B6.129×1(Cg)-Arrb2tmRjl/J, stock no. 023852, The Jackson Laboratory) mice were used. For knockout mice, homozygous offspring (−/−) and wild-type (+/+) littermates from heterozygous breeding pairs were used. For the conditioned place preference (CPP) experiment, male, 8–12-week-old Sprague-Dawley rats were also used. Mice were housed 2–5 per cage while rats were housed 2–3 per cage in the Victoria University of Wellington Small Animal Facility, which was kept at 20–22 °C and 50% humidity. Lights were on from 0700 to 1900 h and testing was conducted only during the light cycle. Food and water were available *ad libitum* except during testing unless specified. Animals were appropriately habituated to the experimental conditions prior to experiments. Tests were conducted by an experimenter blinded to the treatment conditions and protocols were approved by the Victoria University of Wellington Animal Ethics Committee.

### Drug preparation

Kurkinorin and kurkinol were synthesized from salvinorin A as previously described [14, 15]. Morphine sulphate solution (10 mg/mL in 0.9% saline) was purchased from Hospira, New Zealand, β-funaltraxamine (β-FNA) was purchased from Sigma Aldrich, New Zealand, and paclitaxel was purchased from Tocris Bioscience, United Kingdom. All drugs were prepared in a vehicle consisting of dimethyl sulfoxide, tween-80, and 0.9% saline at a ratio of 2:1:7, respectively, except for paclitaxel, which was prepared in a 1:1:18 solution of ethanol, Cremophor, and 0.9% saline. Drugs were administered to mice at a volume of either 5 µL/g subcutaneously (cumulative dose response assays and β-FNA pre-treatment) or 10 µL/g intraperitoneally (all other experiments) and to rats at a volume of 1 mL/kg intraperitoneally.

### Warm-water tail withdrawal

Spinally mediated, thermal antinociception was measured using the warm-water tail withdrawal assay, as previously described [14, 15]. The latency to elicit a tail withdrawal response from a 50 °C water bath was recorded with a maximum latency of 10 seconds. The % maximum possible effect (%MPE) was calculated as follows: %MPE = (test latency – baseline latency) / (maximum latency – baseline latency). To evaluate tolerance, a cumulative dose response procedure was used [6]. On day 1, withdrawal latencies were measured following administration of escalating, incremental doses of drug every 30 min to generate a dose response curve. Morphine (10 mg/kg), kurkinorin (10 mg/kg), or kurkinol (5 mg/kg), which equates to an approximately 2×ED_50_ dose, was then administered once daily from days 2–8, before the dose response experiment was repeated on day 9.

### Hotplate

Supraspinal-mediated thermal antinociception was determined using an incremental HotPlate Analgesia Meter (IITC, Woodland Hills, CA, USA) set to 50 °C as previously described [16]. The time to elicit jumping, licking, or flicking of either hind paw was measured with a 30 second maximum cutoff time. Three baseline measurements were taken, then additional measurements were taken at 15, 30, 45, 60, 90, and 120 min following the administration of MOR agonist. The %MPE was calculated as described above.

### Paclitaxel-induced neuropathic pain and allodynia

Chronic, paclitaxel-induced neuropathic pain was assessed as previously described [17]. During the induction phase (day 0–15), mice were administered paclitaxel (0 or 4 mg/kg) on four alternate days (days 0, 2, 4, and 6). Mechanical and thermal allodynia was measured approximately every other day thereafter. Mechanical allodynia was measured with an electric von Frey apparatus (filament #7, Anesthesiometer 2390 series, IITC Life Science, CA, USA) while thermal allodynia was tested by applying a droplet of acetone to the plantar surface of the hind paw. On day 15, paclitaxel-treated mice were run through a cumulative dose response procedure as described above for both mechanical and thermal allodynia. During the treatment phase (day 17–37), mice were chronically administered MOR agonist, once daily, at ~ED_50_ doses calculated from the mechanical allodynia cumulative dose response on day 15, with allodynia continuing to be measured every other day.

### Whole body plethysmography

Respiratory function was measured using whole body plethysmography based on previous methods [18, 19]. Briefly, a sealed plexiglass chamber connected to an identical reference chamber via a differential pressure transducer, which was connected to a bridge amplifier (PowerLab 26 T, AD instruments, Dunedin, New Zealand) and recordings charted using LabChart8 software (ADInstruments, Dunedin, New Zealand). To calibrate the pressure recordings, 200 µL of air was injected into the chamber. The chamber was kept at 30 °C and a constant humidity by passing carbogen gas (5% O_2_ in CO_2_, BOC, NZ) through a scintillated glass bead humidifier in a 75°C water bath.

Mice were placed into the recording chamber and three baseline measurements were taken prior to the administration of MOR agonist. Recordings were taken at 5-min intervals for 60 min thereafter. Respiratory data from 5 seconds of clean trace (when the mice were not moving) at each time interval was analyzed to calculate the tidal volume and respiratory frequency, which was expressed as a % change from baseline.

### Charcoal meal assay

Mice were fasted for 24 hr and then administered MOR agonist prior to oral gavage of a charcoal meal bolus (5% aqueous suspension of charcoal in a 10% gum arabic solution administered at a volume of 10 μL/g) based on previous methods [4]. Mice were euthanized by CO_2_ asphyxiation 30 min later and the small intestine was dissected. The total length of the small intestine (duodenum to ileum) and the distance to the charcoal meal was measured.

### Metabolic chamber

Mice were administered MOR agonist and placed in a metabolic chamber (Techniplast, NSW, Australia) with free access to water, but not food, for 6 h. The chamber separates feces and urine, which were weighed at hourly intervals.

### Conditioned place preference

Conditioned reward was measured in male rats using an 8-day CPP paradigm exactly as previously described [15]. Conditioned reward was also measured in mice using a 5-day procedure based on previous methods [20, 21]. On day 1, mice were allowed to freely explore a 3-chambered place preference apparatus (PanLab, Harvard Apparatus) for 15 min. The time spent in each chamber was recorded using an overhead camera and video tracking software (SMART v3.0 software, Panlab Harvard Apparatus). On days 2–4, in the morning, mice were administered vehicle and confined to their preferred chamber for 30 min. In the afternoon (4-h later), mice were given MOR agonist and confined to their least preferred chamber for 30 min. On day 5, mice were again allowed to freely roam entire apparatus for 15 min and the percentage change in preference for the drug-paired chamber relative to day 1 was determined.

### Rotarod

Impairment of motor-coordination was assessed using a 5-lane accelerating rotarod (Harvard apparatus, Holliston, MA, USA) set to accelerate from 3–40 rpm over 5 min [15]. After 20 training sessions over 5 days, the latency to fall was measured at baseline (average of three) and at 15, 30, 45, 60, 90, 120, and 180 min following the administration of MOR agonist.

### Statistical analysis

A detailed summary of all statistical analyses and test results can be found in supplementary Table S1. All statistical analyses were carried out with GraphPad prism (v10.1.0) or SPSS (v28.0.1.0). Results were considered statistically significant when *p* < 0.05. There were few significant differences between male and female mice, therefore these data were pooled for the main analyses. Un-pooled data with sex included as a factor in the analyses are described in the supplementary materials, Figs. S1–5, and Table S2.

## Results

### Kurkinorin and kurkinol showed MOR-dependent antinociceptive effects

Previously, we reported the synthesis of two novel, selective agonists of the MOR, kurkinorin and kurkinol (Fig. 1A) that had significant antinociceptive effects in the warm-water tail withdrawal assay, which primarily measures spinally mediated antinociception [14, 15]. To expand on these findings, we first examined the supra-spinally mediated antinociceptive effects of these drugs using the hotplate assay (Fig. 1B–F). Morphine and kurkinorin had a similar duration of action while the effects of kurkinol were shorter (Fig. 1B). All compounds had a significant overall effect at both low (~ED_50_; Fig. 1C) and high doses (~2×ED_50_; Fig. 1D). The antinociceptive effects of morphine, kurkinorin, and kurkinol in both the hot plate (Fig. 1E, F) and tail withdrawal (Fig. 1G) assay were abolished in MOR knockout mice, confirming that these effects were due to activity at the MOR.

### Reduced βArr2 signaling increased antinociceptive potency but did not prevent the development of tolerance

To investigate the role of βArr2 recruitment in the antinociceptive effects of MOR agonists, the potency (ED_50_) of morphine, kurkinorin, and kurkinol was determined using a cumulative dose-response procedure before and after 7 days of treatment with a ~ 2×ED_50_ dose in βArr2 knockout mice (Fig. 1H–J). In drug-naïve wild-type mice, the rank order of potency was kurkinol > kurkinorin > morphine. When retested following 7 days of drug treatment, there was a rightward shift in the dose response curve for both morphine (Fig. 1H) and kurkinorin (Fig. 1I), with significantly greater ED_50_ values indicating tolerance. Interestingly, kurkinol-treated mice showed no significant evidence of tolerance (Fig. 1J). Knockout of βArr2 significantly increased the potency of both morphine (Fig. 1H) and kurkinorin (Fig. 1I) but did not prevent the development of tolerance. Knockout of βArr2 had no significant impact on the effects of kurkinol (Fig. 1J).

### Kurkinorin and kurkinol attenuated neuropathic pain but developed tolerance similar to morphine

**Fig. 2:**
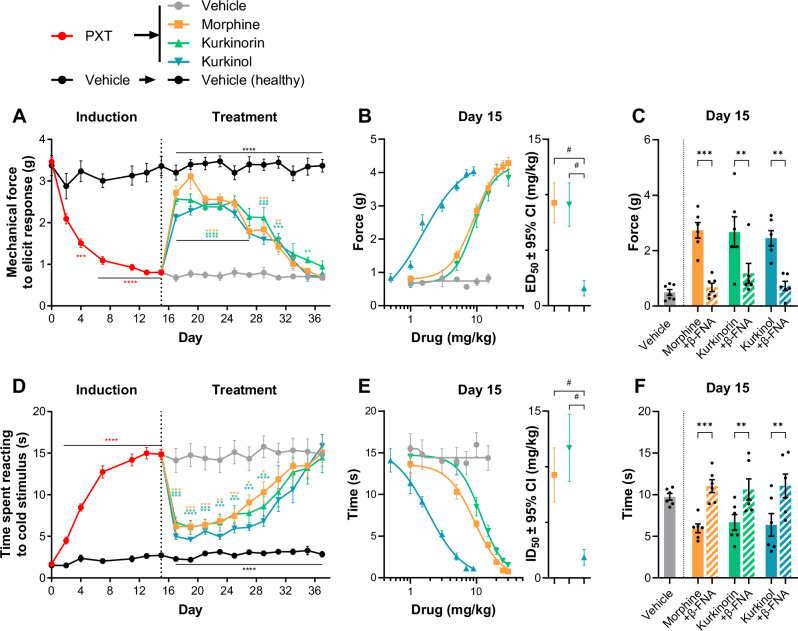
Morphine, kurkinorin, and kurkinol all similarly reverse paclitaxel-induced allodynia in a MOR-dependent manner but show significant tolerance with repeated treatment. Paclitaxel (4 mg/kg) was administered on day 0, 2, 4 and 6 to induce both mechanical **A** and thermal **D** allodynia in male and female mice (pooled), which were subsequently treated with daily administration of ED_50_ doses of morphine, kurkinorin, or kurkinol from day 17–37. Cumulative dose response curves for each MOR agonist and calculated ED_50_ values (± 95% confidence intervals) for mechanical **B** and thermal **E** allodynia on day 15. Effect of pre-treatment with the MOR antagonist, β-FNA (5 mg/kg), 24 h prior to MOR agonist administration and mechanical **C** and thermal **F** allodynia testing in a separate cohort of male mice. All data are presented as mean ± SEM unless otherwise stated. *n* = 11–12/treatment **A, B, D, E**, *n* = 6/treatment **C, F**. *p < 0.05, **p < 0.01, ***p < 0.001, ****p < 0.0001, compared to vehicle treatment or as indicated, two-way ANOVA. ^#^95% confidence intervals.

Next, we evaluated the effects of kurkinorin and kurkinol on paclitaxel-induced neuropathic pain (Fig. 2). During the induction phase, paclitaxel administration rapidly induced chronic mechanical (Fig. 2A) and thermal (Fig. 2D) allodynia. Following the induction, on day 15, cumulative dose response curves indicated that the rank order of potency was kurkinol > kurkinorin = morphine in both the mechanical (Fig. 2B) and thermal (Fig. 2E) allodynia tests. During the treatment phase, daily administration of ~ED_50_ doses of MOR agonist significantly attenuated both mechanical (Fig. 2A) and thermal (Fig. 2D) allodynia initially, but all drugs failed to have a significant effect by day 37, indicating tolerance. In a separate cohort of paclitaxel-inducted mice, the MOR antagonist, β-FNA, blocked the ability of morphine, kurkinorin, or kurkinol to reverse mechanical (Fig. 2C) and thermal allodynia (Fig. 2F) indicating that these effects are MOR-dependent.

### Kurkinorin and kurkinol induced respiratory depression similar to morphine and knockout of βArr2 had no effect

**Fig. 3:**
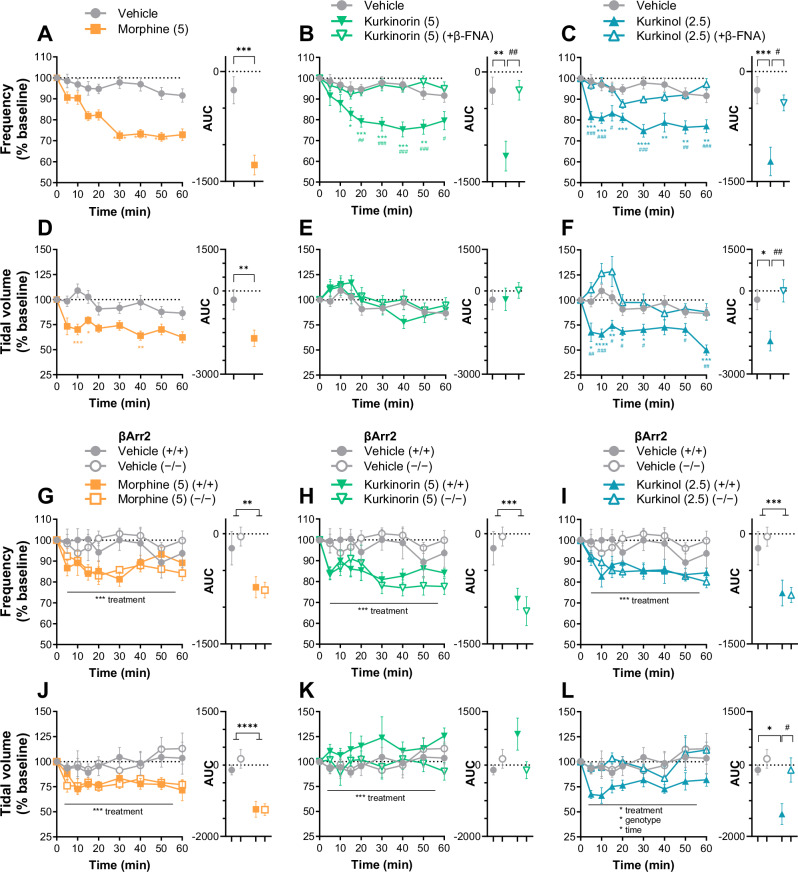
Morphine, kurkinorin, and kurkinol cause MOR-dependent respiratory depression, as determined by whole body plethysmography, and knockout of βArr2 has little effect. Time-dependent and overall (AUC) effect of morphine, kurkinorin, and kurkinol on respiratory frequency **A–C** and tidal volume **D–F** in male and female mice (pooled) pre-treated with the MOR antagonist, β-FNA (5 mg/kg), 24 h prior (kurkinorin and kurkinol only). Time-dependent and overall (AUC) effect of morphine, kurkinorin, and kurkinol on respiratory frequency **G–I** and tidal volume **J–L** in βArr2 knockout male mice. Drug doses are mg/kg. All data are presented as mean ± SEM. *n* = 11–16/treatment **A–F**, *n* = 5–7/treatment/genotype **G–L**. *p < 0.05, **p < 0.01, ***p < 0.001, ****p < 0.0001, compared to vehicle treatment, one or two-way ANOVA. ^#^*p* < 0.05, ^##^*p* < 0.01, ^###^*p* < 0.001, ^####^*p* < 0.0001 compared to β-FNA-treatment or βArr2 knockout, one or two-way ANOVA.

Next, whole-body plethysmography was carried out to determine the effect of the MOR agonists on respiration (Fig. 3). Morphine (Fig. 3A), kurkinorin (Fig. 3B), and kurkinol (Fig. 3C) all significantly reduced respiratory frequency to a similar extent over a 60-min period relative to vehicle-treated controls. Interestingly, only morphine (Fig. 3D) and kurkinol (Fig. 3F) significantly reduced tidal volume, whereas kurkinorin (Fig. 3E) had no significant effect. However, a closer examination reveals that kurkinorin caused a significant reduction in tidal volume in female but not male mice (Fig. S3E). All significant effects of morphine, kurkinorin, and kurkinol were reversed by pre-treatment with the MOR antagonist, β-FNA (Fig. 3B, C, F; Fig. S3).

Since βArr2 has been implicated not only in the antinociceptive effects of MOR agonists but also their side effects, we then repeated this study with βArr2 knockout mice. In wild-type controls, results mimicked those described above (Fig. 3G–L). Knockout of βArr2 largely had no effect: respiratory frequency and tidal volume did not significantly change as a function of genotype in any treatment condition (Fig. 3G–K), except for kurkinol-treated mice, where knockout of βArr2 appeared to reverse the effect of kurkinol on tidal volume (Fig. 3L).

### Kurkinorin and kurkinol reduced gastrointestinal transit similar to morphine and knockout of βArr2 had no effect

**Fig. 4:**
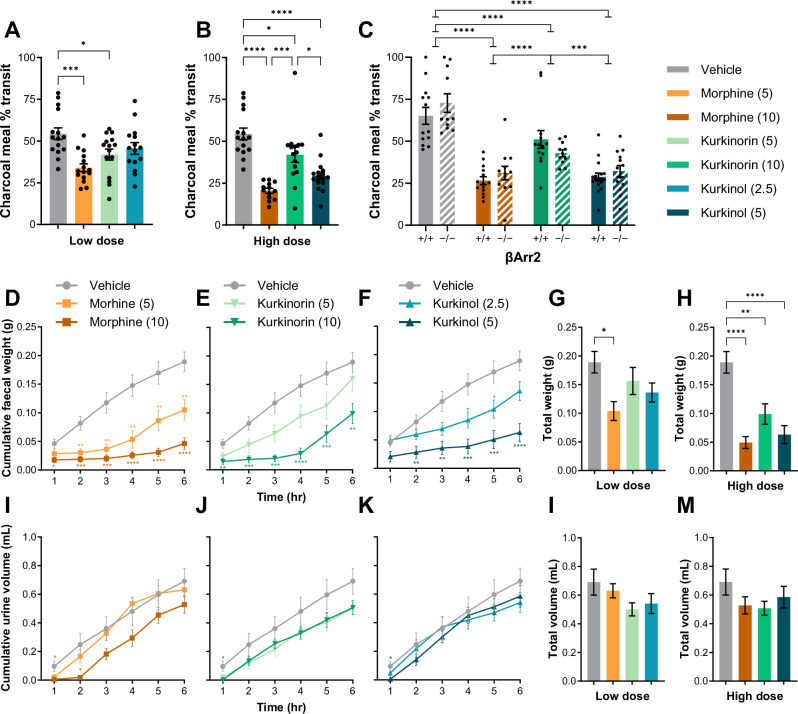
Morphine, kurkinorin, and kurkinol have similar effects on gastrointestinal transit in mice that is not impacted by knockout βArr2. Effect of administration of low **A** or high **B** doses of morphine, kurkinorin, and kurkinol on percent small intestinal transit of a charcoal meal bolas in male and female mice (pooled). **C** Effect of βArr2 knockout on percent small intestinal transit of a charcoal meal bolas following administration of high doses of morphine, kurkinorin, and kurkinol in male and female mice (pooled). Dose-dependent effects of morphine, kurkinorin, and kurkinol on fecal weight **D–H** or urine volume **I–M** while in a metabolic chamber in male and female mice (pooled). Drug doses are mg/kg. All data are presented as mean ± SEM. *n* = 12–17/treatment. *p < 0.05, **p < 0.01, ***p < 0.001, ****p < 0.0001, compared to vehicle treatment or as indicated, one or two-way ANOVA.

The charcoal meal assay was used to assess small intestinal transit (Fig. 4A–C). At low doses, morphine produced the greatest reduction in intestinal transit (Fig. 4A). At higher doses, all compounds produced a significant reduction, but the effect of kurkinorin was significantly less severe (Fig. 4B). Repeating the experiment in βArr2 knockout mice produced similar results, with no effect of βArr2 knockout (Fig. 4C).

To further investigate the gastrointestinal as well as the urinary retentive effects of our MOR agonists, the accumulation of feces and urine was separated using a metabolic chamber and recorded for 6 h following the administration of morphine, kurkinorin, or kurkinol (Fig. 4D–M). Morphine significantly reduced fecal accumulation at both low and high doses (Fig. 4D, G, H) while kurkinorin (Fig. 4E, G, H) and kurkinol (Fig. 4F–H) primarily had significant effects only at higher doses. A small, but significant reduction in urine volume was measured 1–2 h after morphine administration (Fig. 4I), and 1 h after kurkinorin (Fig. 4J) and kurkinol (Fig. 4K) administration, but otherwise urine output was unaffected (Fig. 4L, M).

### Kurkinol caused motor impairment similar to morphine, but with a shorter duration of action

**Fig. 5:**
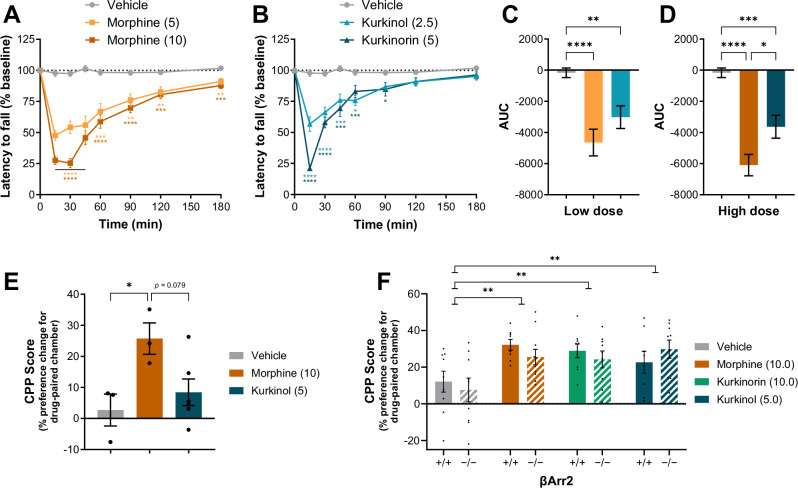
Kurkinol has similar motor impairing effects to morphine but with a shorter duration of action while the conditioned rewarding effects of morphine, kurkinorin, and kurkinol are not impacted by knockout of βArr2 in mice. Time dependent **A, B** and overall (AUC; **C, D**) effects of morphine and kurkinol on the latency to fall from an accelerating rotarod in male and female mice (pooled). **E** Effect of morphine and kurkinol on conditioned place preference score (% change in preference for drug-paired chamber) in male rats. **F** Effect of morphine, kurkinorin, and kurkinol on conditioned place preference score in βArr2 knockout male and female mice (pooled). Drug doses are mg/kg. All data are presented as mean ± SEM. *n* = 12–14/treatment **A–D**, *n* = 3–6/treatment **E**, *n* = 8–10/treatment **F**. *p < 0.05, **p < 0.01, ***p < 0.001, ****p < 0.0001, compared to vehicle treatment or as indicated, one or two-way ANOVA.

We have previously shown that kurkinorin produced a shorter duration of motor impairment on the rotarod assay compared to morphine [14]. Therefore, here we compared the effects of kurkinol (Fig. 5A–D). Morphine produced significant impairment for at least 180 min (Fig. 5A) while kurkinol had a significant effect for up to 90 min (Fig. 5B). Kurkinol produced significantly less overall motor impairment at both low (Fig. 5C) and high doses (Fig. 5D) relative to morphine, though this may be due to pharmacokinetic factors since kurkinol also had a shorter duration of action in the tail-withdrawal [14] and hotplate assays (Fig. 1).

### All drugs showed conditioned reinforcing effects in mice and knockout of βArr2 had no effect

We have also previously shown that, unlike morphine, kurkinorin did not produce a significant rewarding effect in rats in the CPP paradigm [15]. Therefore, here we determined the rewarding effects of kurkinol in rats in the same manner (Fig. 5E). Morphine significantly increased CPP score (% change in preference for the drug paired chamber post conditioning), indicating a rewarding effect, whereas kurkinol had no significant effect (Fig. 5E). We then repeated this study with morphine, kurkinorin, and kurkinol in a large sample of wild-type and βArr2 knockout mice (Fig. 5F). Here, all drugs produced a significant conditioned rewarding effect compared to vehicle-treated controls while knockout of βArr2 had no significant impact on the conditioned rewarding effects of any MOR agonist.

## Discussion

### Role of βArr2 signaling in MOR agonist anticonception and tolerance

It has been suggested that reduced activation of βArr2 signaling pathways by MOR agonists may enhance their analgesic effects and reduce the development of tolerance. Evidence for this idea has come from several studies showing that genetic deletion, downregulation, or inhibition of βArr2 signaling can enhance the antinociceptive effects of morphine and attenuate the development of tolerance in the hotplate and tail withdrawal assays [7–10, 22–25]. Not all findings have been consistent, however, and the role of βArr2 signaling may be ligand dependent. For example, knockout of βArr2 had no effect on the potency of fentanyl, methadone, or oxycodone and did not impact the development of tolerance to these drugs [25]. In contrast, another study using mice expressing a phosphorylation-deficient MOR to inhibit β-arrestin recruitment (both 1 and 2 isoforms) found reduced antinociceptive tolerance to morphine and fentanyl [23]. These discrepancies highlight the potential for compensatory signaling by β-Arrestin1 in βArr2 knockout models.

Our findings provide some support for the idea that βArr2 is involved in opioid-induced analgesia and tolerance. In the tail withdrawal assay, knockout of βArr2 resulted in an increase in antinociceptive potency for morphine and kurkinorin, but did not prevent the development of tolerance. Interestingly, knockout of βArr2 had no impact on the antinociceptive effects of kurkinol, which might be due to kurkinol having little impact on βArr2 ordinarily due to its greater G-protein signaling bias. We also found that, kurkinol, our most G-protein biased agonist, was the most potent drug in all antinociceptive tests and showed no significant tolerance in the tail withdrawal assay after 7 days of ~2×ED_50_ dosing, which is consistent with prior studies showing limited antinociceptive tolerance to other G-protein biased MOR agonists, including TRV-130/oliceridine [26] and mitragynine pseudoindoxyl [27]. Though it should be mentioned that the use of different assays, cell lines, receptor species, reference ligands, and experimental models make it difficult to reliably compare G-protein signaling bias across studies [11, 12, 28]. Interestingly, all of our drugs, including kurkinol, showed similar tolerance in the chronic paclitaxel-induced neuropathic pain model after several weeks of treatment.

These results suggest that although some agonists may show reduced tolerance in acute pain models, such as the tail withdrawal assay, which acts on an undamaged spinothalamic pain pathway, this does not translate to more chronic models such as paclitaxel-induced peripheral neuropathy. Paclitaxel causes microtubule dysfunction and impedes axonal transport, leading to peripheral nerve fiber damage, demyelination, and inflammation, as well as a dysregulation of Ca2+ signaling and altered circuitry throughout the peripheral and central nervous systems [29, 30]. In this damaged and dysregulated system, the mechanisms typically thought to moderate MOR-mediated antinociception and tolerance/receptor desensitization such as voltage-gated calcium channel coupling, β-Arrestin recruitment, and phosphorylation of G-protein couple receptor kinase and extracellular signal-regulated kinases 1/2 may be altered [31–36]. This highlights the importance of evaluating therapeutic viability of novel compounds and investigating the role of signaling bias in multiple pain models and in the particular disease state of interest.

### Role of βArr2 signaling in MOR agonist side effects

Initial research on MOR-induced side effects in βArr2 knockout mice suggested that βArr2 signaling may mediate their respiratory and gastrointestinal effects [4]. However, several recent studies in βArr2 knockout mice or knock-in mice with a MOR-specific mutation to prevent βArr2 recruitment failed to replicate these findings with morphine and other mu opioids [23, 37–42]. Research into MOR-induced side effects using novel G-protein biased agonists has also produced mixed results. For example, TRV130 and PZM21 have been shown to produce both less [43–45] as well as equal or more [26, 46, 47] respiratory depression and constipation compared to morphine at equianalgesic doses, though their G-protein bias has also been questioned [12, 48]. In a study using a range of novel MOR agonists (SR compounds), increased G-protein bias correlated with reduced respiratory depression [3]. In contrast, another study found that reduced respiratory depression did not correlate with G-protein bias, but with lower intrinsic efficacy [49], which led to the suggestion that it is lower efficacy agonists of the MOR that have a more favorable side effect profile [12, 42, 48].

In the current study, we showed that morphine, kurkinorin, and kurkinol, all produced similar significant decreases in respiratory frequency, despite their varying G-protein bias. Morphine and kurkinol also produced similar decreases in tidal volume, whereas this effect was only produced by kurkinorin in female mice. Sex differences in opioid-induced respiratory depression are not well-understood [50–52], but the presence of a drug- and sex-specific effect highlights the importance of evaluating novel compounds in both male and female mice. We found that knockout of βArr2 had no effect on respiratory depression (frequency or tidal volume) caused by any drug, with the curious exception of reversing kurkinol-induced decreases in tidal volume. These results largely support the recent consensus that βArr2 signaling does not mediate MOR-induced respiratory depression [11, 12, 48].

Also consistent with recent conclusions [11, 12, 48], our results suggest that MOR-induced constipation is similarly not mediated by βArr2 signaling. Morphine, kurkinorin, and kurkinol all produced decreases in small intestinal transit and overall fecal output, with the least G-protein biased compound, kurkinorin, having the smallest effect on both measures. Moreover, knockout of βArr2 had no impact on small intestinal transit in vehicle- or MOR-agonist treated mice.

We also investigated the role of βArr2 signaling in conditioned reward. Reduced abuse liability is a critical feature to achieve with novel MOR agonists, since it is the reinforcing effects of these drugs drives their misuse, which in turn, can result in a lethal overdose. Previous research has shown that knockout of βArr2 in mixed C57BL/6 × 129SvJ mice enhanced the rewarding effects of morphine in the CPP paradigm and increased extracellular dopamine release in the striatum, suggesting that G-protein biased MOR agonists may actually have greater abuse liability [53]. Other studies using various G-protein biased agonists have shown mixed results. TRV130, PZM21, and mitragynine pseudoindoxyl did not induce significant place preference [27, 44, 45]. However, as previously mentioned, the status of PZM21 as a G-protein biased agonist has been challenged [46], and mitragynine pseudoindoxyl also has activity at other opioid receptors, which may alter its in vivo effects [27]. Additional studies that used drug-discrimination, intracranial self-stimulation, or self-administration procedures showed that TRV130 has subjective and reinforcing effects similar to other abused opioids such as morphine, oxycodone, and fentanyl [26, 54, 55]. PZM21 has also been shown to be self-administered in non-human primates at similar rates to oxycodone [56]. Here we showed that knockout of βArr2 had no impact on CPP in mice induced by any MOR agonist tested, suggesting that βArr2 signaling plays no role in mediating MOR-induced conditioned reward. Interestingly, we previously showed that kurkinorin did not produce significant CPP in rats [15] and here we showed the same result with kurkinol, albeit with a small sample size. When evaluated in a large sample of mice, however, both compounds produced a significant conditioned rewarding effect, which could be due to dosing differences between rats and mice.

### Conclusions

While some recent findings have suggested that G-protein biased MOR agonists may have increased therapeutic windows, these findings are largely correlational and could be driven by numerous other factors. To better identify the causal role of βArr2 signaling in the antinociceptive and side effects of MOR agonists we carried out a series of experiments using βArr2 knockout mice. We found that knockout of βArr2 can result in a drug-specific increase in antinociceptive potency. However, knockout of βArr2 largely had no impact on MOR-induced respiratory depression, constipation, conditioned reward, or antinociceptive tolerance suggesting a limited role of βArr2 signaling. As part of these experiments, we also found that the novel MOR agonists tested in this study, kurkinol and kurkinorin, show some therapeutic improvements over morphine that may warrant further investigation.

## Supplementary information

Supplimentary tables

Effects of Sex

## Data Availability

Data are available from the corresponding author upon reasonable request.
