# Supplementary material for: Role of β-Arrestin 2 in the antinociceptive and side effect profile of morphine and the novel mu opioid receptor agonists, kurkinorin and kurkinol
Source: Neuropsychopharmacology. 2025 Sep 3;50(12):1777–86. doi: 10.1038/s41386-025-02214-z (PMC12518651; doi:10.1038/s41386-025-02214-z)
Supplement: Supplementary file 2 — Effects of Sex [file 41386_2025_2214_MOESM2_ESM.pdf]

SUPPLEMENTARY MATERIAL FOR:

Role of β-Arrestin 2 in the antinociceptive and side effect profile of morphine and the novel mu opioid receptor agonists, kurkinorin and kurkinol.

Ross van de Wetering, PhD[1]

Amy F. Alder, PhD[1]

Andrew Biggerstaff, PhD[1]

Katya Sellen, MSc[1]

Dan Luo, PhD[2]

Rachel S. Crowley, PhD[3]

Thomas E. Prisinzano, PhD[2]

Bronwyn M. Kivell, PhD[1*]

[1]School of Biological Sciences, Centre for Biodiscovery, Victoria University of Wellington, Wellington 6012, New Zealand.

[2]Department of Pharmaceutical Sciences, University of Kentucky, Lexington, Kentucky 40506, United States.

[3]Department of Medicinal Chemistry, School of Pharmacy, The University of Kansas, 1251 Wescoe Hall Drive, 4070 Malott, Lawrence, Kansas 66045, United States.

[*]Corresponding author

Table S1 and S2 – Statistical analyses and results – see attached excel table

Effects of Sex

Splitting the hotplate data by sex, there were a handful of significant differences between male and female mice (**Figure S1**). Both morphine (**Figure S1A**) and kurkinorin (**Figure S1A**) had a slightly different time course of effect as a function of sex and the overall effect of a low dose of kurkinorin was significantly less in female mice compared to male mice (**Figure S1D**).

There were no significant differences in paclitaxel-induced mechanical (**Figure S2A**) or thermal (**Figure S2B**) allodynia between male and female mice. During the treatment phase, there was a small but significant decrease in the potency of kurkinorin in female mice compared to males in the mechanical allodynia tests (**Figure S2C**), but otherwise sex had no significant impact on the effect of drug treatment in these experiments (**Figure S2**).

There were few significant differences between male and female mice in MOR-induced respiratory depression as a function of time but no overall significant differences (**Figure S3**) with the interesting exception of kurkinorin appearing to significantly decrease tidal volume in female mice, but not male mice (**Figure S3E**).

There were no significant differences in small intestinal transit between male and female mice (**Figure S4A-C**). The effect of kurkinorin and kurkinol on reduced fecal output was significantly greater in female mice compared to males (**Figure S4D-H**). Total urine volume was significantly greater in vehicle-treated male mice compare to females (**Figure S4I-M**), with morphine and kurkinorin treatment both significantly decreasing urine volume in males but not females (**Figure S4L-M**).

When comparing between male and female mice on the accelerating rotarod (**Figure S5A-D**), the low dose of morphine produced slightly less motor impairment in female mice while the low dose of kurkinol caused slightly more motor impairment in female mice, but otherwise there were no significant effects of sex. There was no significant interaction or main effects of sex in MOR agonist-induced conditioned place preference (**Figure S5E**).

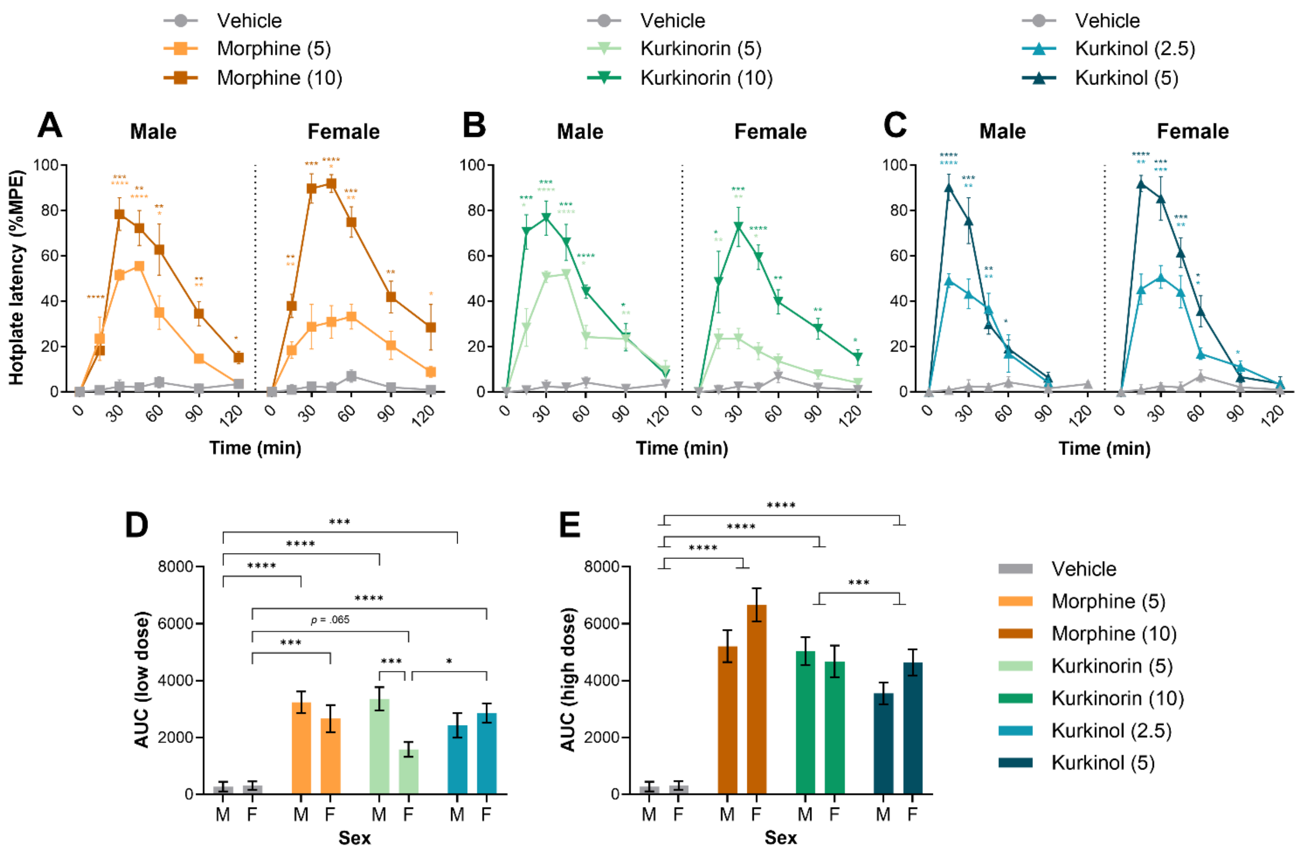

Figure S1. Time-dependant (**A-C**) and overall (AUC; **D-E**) antinociceptive effects of morphine, kurkinorin, and kurkinol in the hotplate assay in male and female mice. Drug doses are mg/kg. Data are presented as mean ± SEM unless. n = 5-6/treatment/sex. *p < .05, **p < .01, ***p < .001, ****p < .0001 compared to vehicle treatment or as indicated, two or three-way ANOVA.

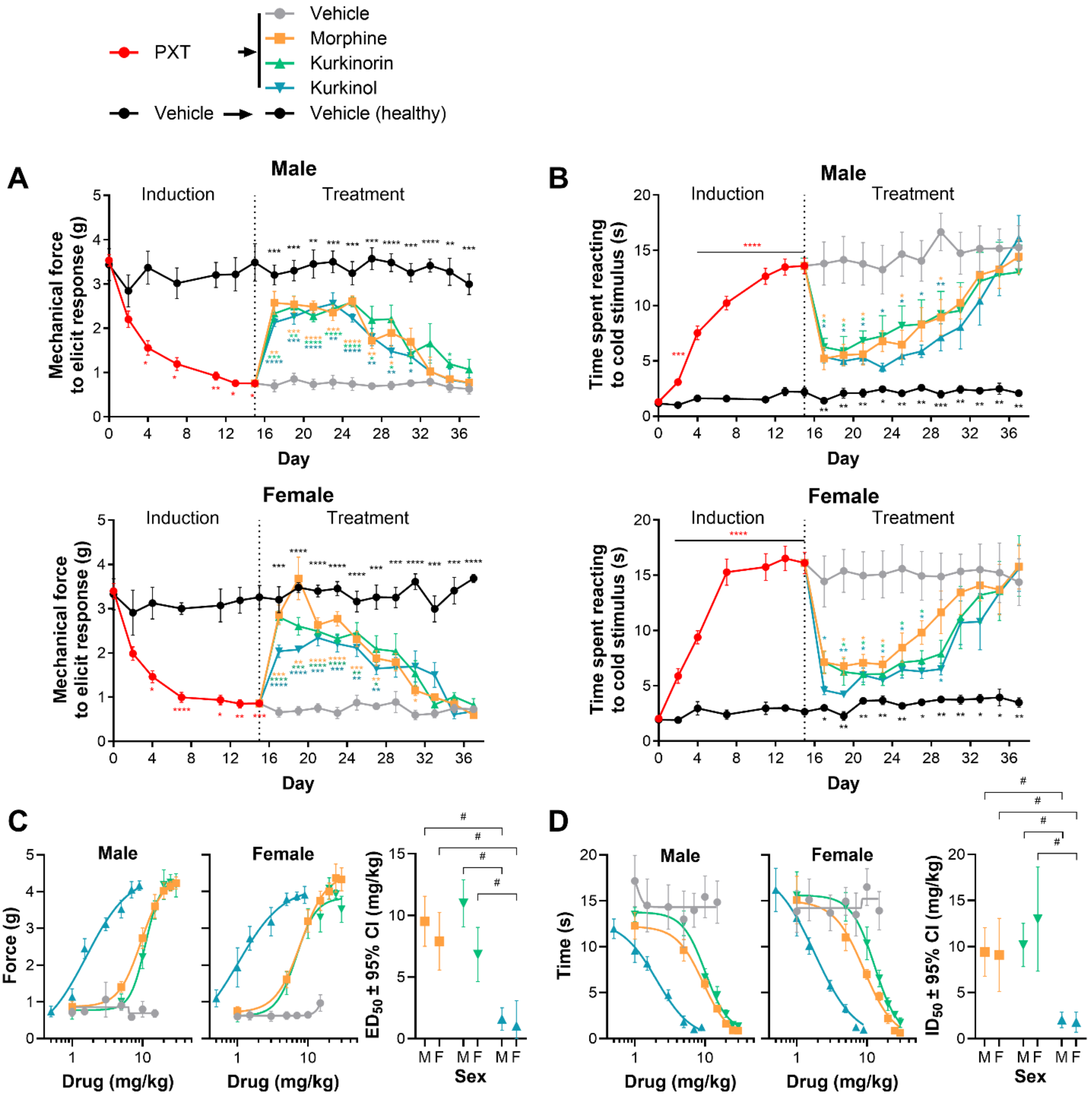

Figure S2. Paclitaxel (4 mg/kg) was administered on day 0, 2, 4 and 6 to induce both mechanical (**A**) and thermal (**B**) allodynia in male and female mice, which were subsequently treated with daily administration of ED_{50} doses of morphine, kurkinorin, or kurkinol from day 17-37. Cumulative dose response curves for each MOR agonist and calculated ED_{50} values (± 95% confidence intervals) for mechanical (**C**) and thermal (**D**) allodynia on day 15. All data are presented as mean ± SEM unless otherwise stated. n = 5-6/treatment/sex. *$p < .05$, **$p < .01$, ***$p < .001$, ****$p < .0001$, compared to vehicle treatment, three-way ANOVA. [#]95% confidence intervals.

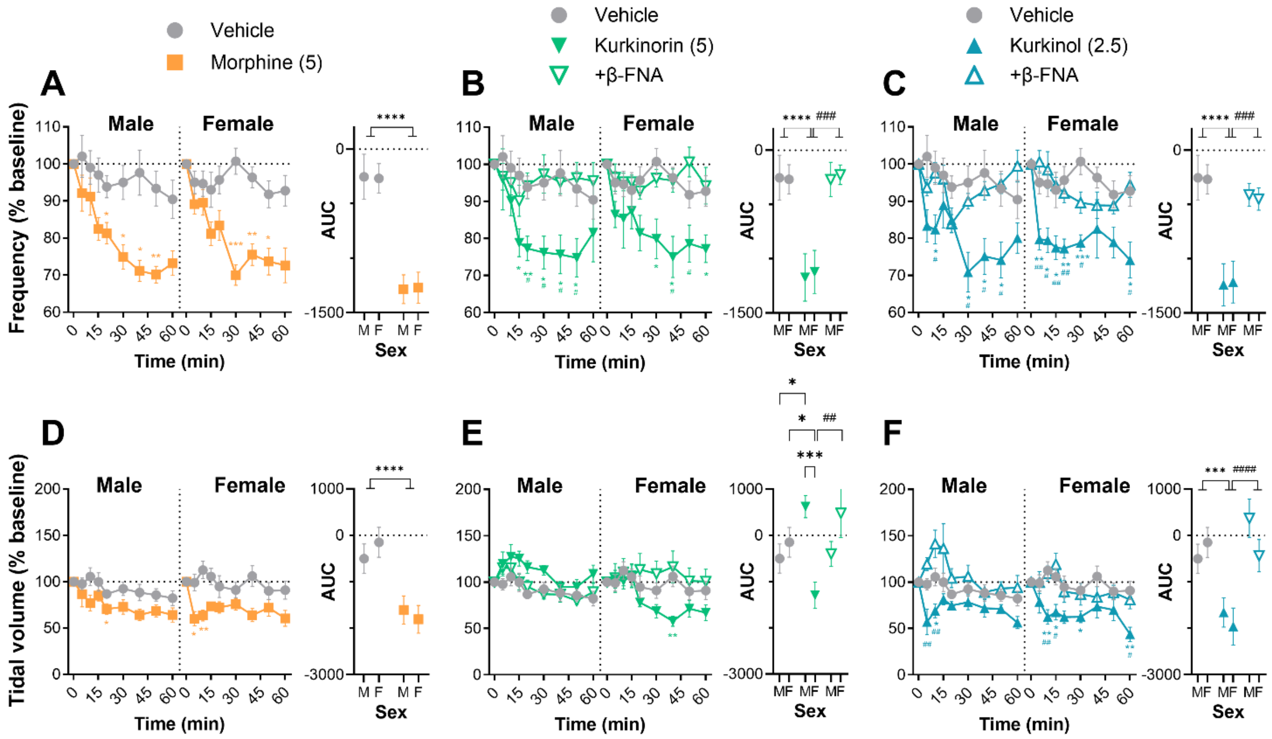

Figure S3. Time-dependent and overall (AUC) effect of morphine, kurkinorin, and kurkinol on respiratory frequency (**A-C**) and tidal volume (**D-F**) as determined by whole body plethysmography in male and female mice pre-treated with the MOR antagonist, β-FNA (5 mg/kg), 24 hours prior (kurkinorin and kurkinol only). Drug doses are mg/kg. All data are presented as mean ± SEM. n = 5-8/treatment/sex. *p < .05, **p < .01, ***p < .001, ****p < .0001, compared to vehicle treatment or as indicated, two or three-way ANOVA. $^{\#}p$ < .05, $^{\#\#}p$ < .01, $^{\#\#\#}p$ < .001, $^{\#\#\#\#}p$ < .0001 compared to β-FNA-treatment, two or one-way ANOVA.

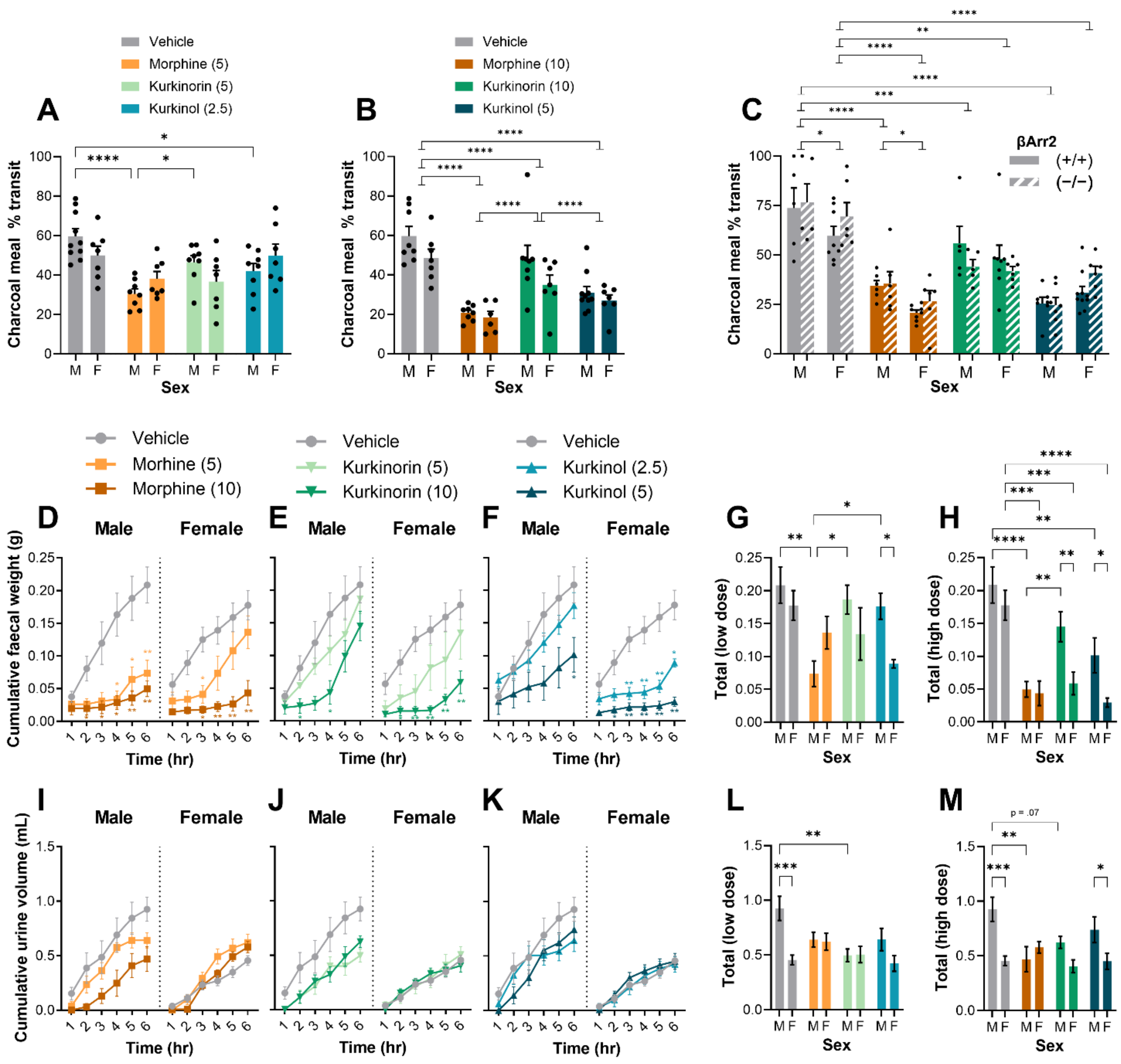

Figure S4. Effect of low (**A**) or high (**B**) doses of morphine, kurkinorin, and kurkinol on percent small intestinal transit of a charcoal meal bolas in male and female mice. (**C**) Effect of βArr2 knockout of percent small intestinal transit of a charcoal meal bolas following administration of high doses of morphine, kurkinorin, and kurkinol in male and female mice. Dose-dependent effects of morphine, kurkinorin, and kurkinol on cumulative faecal weight (**D-H**) or urine volume (**I-M**) while in a metabolic chamber in male and female mice. Drug doses are mg/kg. All data are presented as mean ± SEM. n = 5-10/treatment/sex/genotype. *$p < .05$, **$p < .01$, ***$p < .001$, ****$p < .0001$, compared to vehicle treatment or as indicated, two or three-way ANOVA.

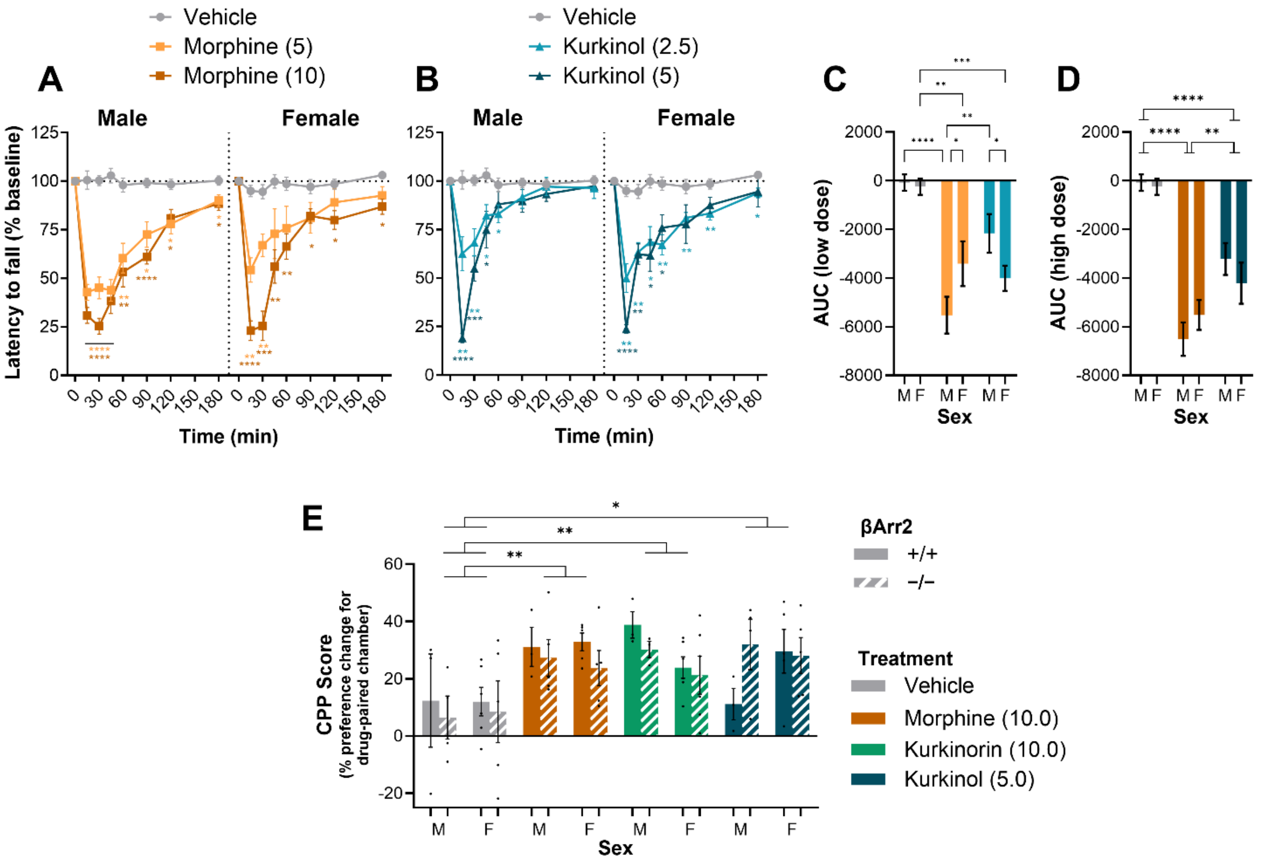

Figure S5. Time dependant (**A-B**) and overall (AUC; **C-D**) effect of morphine and kurkinol on the latency to fall from an accelerating rotarod in male and female mice. (**E**) Effect of morphine, kurkinorin, and kurkinol on conditioned place preference score in βArr2 knockout male and female mice. Drug doses are mg/kg. All data are presented as mean ± SEM. n = 5-7/treatment/sex (A-D), n = 3-6/treatment/genotype/sex (E). *$p < .05$, **$p < .01$, ***$p < .001$, ****$p < .0001$, compared to vehicle treatment or as indicated, two or three-way ANOVA.
